# EZHIP in Pediatric Brain Tumors: From Epigenetic Mimicry to Therapeutic Vulnerabilities

**DOI:** 10.3390/ijms27020963

**Published:** 2026-01-18

**Authors:** Tiziana Servidei, Serena Gentile, Alessandro Sgambato, Antonio Ruggiero

**Affiliations:** 1 Pediatric Oncology Unit, Fondazione Policlinico Universitario Agostino Gemelli IRCCS, 00168 Rome, Italy; serena.gentile@unicatt.it; 2 Department of Translational Medicine and Surgery, Università Cattolica del Sacro Cuore, 00168 Rome, Italy; alessandro.sgambato@unicatt.it; 3 Multiplex Spatial Imaging Facility, Fondazione Policlinico Universitario Agostino Gemelli IRCCS, 00168 Rome, Italy; 4 Department of Woman and Child Health and Public Health, Università Cattolica del Sacro Cuore, 00168 Rome, Italy

**Keywords:** EZHIP, EPN-PFA, DMG, H3-altered, PRC2, epigenetics, H3K27M, intrinsically disordered protein

## Abstract

Enhancer of zeste homologs inhibitory protein (EZHIP) is a eutherian-specific protein, with poorly defined developmental functions and physiological expression restricted to germ cells. Its aberrant re-expression characterizes posterior fossa ependymoma subtype A and a subset of diffuse midline gliomas with wild-type histone H3—aggressive pediatric brain tumors marked by global loss of the repressive H3 lysine 27 trimethylation (H3K27me3). Functionally analogous to the H3 lysine 27 to methionine (H3K27M) oncohistone, EZHIP inhibits Polycomb repressive complex 2 (PRC2), altering genome-wide H3K27me3 distribution and fate commitment. Unlike H3K27M, EZHIP is epigenetically silenced under physiological conditions yet inducible, suggesting context-dependent oncogenic roles. Its intrinsically disordered structure enables multifunctional interactions and biological versatility. Beyond brain tumors, EZHIP has emerged as an oncogenic driver in osteosarcoma, underscoring broader relevance across cancers. This review integrates current insights into EZHIP—from gene discovery and the mechanism of PRC2 inhibition to its emerging roles in metabolism, DNA repair, 3D chromatin regulation, and development. We outline EZHIP’s clinico-pathological significance in pediatric and adult malignancies, with an emphasis on EZHIP-driven hindbrain tumors. Finally, we discuss therapeutic opportunities, from the direct targeting of intrinsically disordered proteins to the indirect modulation of EZHIP-associated epigenetic and metabolic landscapes, highlighting implications for tumor evolution and precision oncology.

## 1. Introduction

Posterior fossa ependymoma subtype A (EPN-PFA) and diffuse midline glioma (DMG), H3K27-altered, represent two highly aggressive central nervous system (CNS) malignancies recognized in the fifth CNS WHO Classification by profound epigenetic dysregulation [1]. Although histologically distinct, these entities share a unifying epigenetic hallmark: global loss of histone 3 lysine 27 trimethylation (H3K27me3), a repressive chromatin mark required for normal neurodevelopment [2,3]. H3K27me3 loss results from two mutually exclusive molecular mechanisms: overexpression of enhancer of zeste homologs inhibitory protein (EZHIP) [4], which occurs in the vast majority of EPN-PFAs and in a small subset of DMGs, and the H3 lysine 27 to methionine (H3K27M) oncohistone mutation [5,6,7], which defines most DMGs and is only rarely found in EPN-PFAs [8,9]. Both lesions functionally act as dominant-negative inhibitors of enhancer of zeste 2 Polycomb repressive complex 2 subunit (EZH2), the histone methyltransferase core of the Polycomb repressive complex 2 (PRC2), resulting in altered genome-wide H3K27me3 distribution and the deregulation of developmental and tumor suppressor gene networks [10,11] (for details see Section 2.2).

Clinically, these tumors occur in early childhood and exhibit marked resistance to current treatment strategies with dismal prognoses. Anatomically, they arise from neighboring midline structures in the posterior fossa and brainstem, which are both embryologically derived from the hindbrain lining the fourth ventricle [12]. While most DMG H3K27-altered are located in the pons, which forms the floor of the fourth ventricle, EPN-PFA typically originates dorsally, from the roof, near the cerebellum. This spatial patterning suggests that region-specific neural stem/progenitor cells—oligodendroglial progenitors in DMG or radial glial cells in EPN-PFA—are selectively vulnerable to PRC2 dysregulation during restricted developmental windows, leading to altered cell fate specification, stalled differentiation, and malignant transformation. The intricate interplay between an epigenetic event, developmental timing, and the cell of origin has been reviewed in detail very recently [13].

While the role of oncohistones in pediatric brain tumors is well-established [14,15,16,17], the function(s) of EZHIP remains comparatively far less defined. EZHIP is a non-histone, intrinsically disordered protein with physiological expression largely confined to germ cells and epigenetic silencing in most somatic tissues [18]. Its aberrant reactivation in pediatric hindbrain tumors suggests a context-dependent oncogenic role that likely extends beyond simple PRC2 inhibition. In this review, we summarize the current knowledge of EZHIP, from its gene and protein features to its mechanisms of PRC2 inhibition and emerging non-canonical functions in chromatin organization, metabolism, and DNA damage response. We integrate data from cellular, animal, and developmental models and examine EZHIP expression across pediatric and adult malignancies, focusing on its clinico-pathological relevance in EPN-PFA and DMG H3 wild-type. Finally, we discuss potential therapeutic strategies aimed at exploiting EZHIP-associated vulnerabilities, with implications for precision approaches in pediatric neuro-oncology.

## 2. EZHIP: A Protein in Its Infancy

### 2.1. The EZHIP Gene

**Figure 1:**
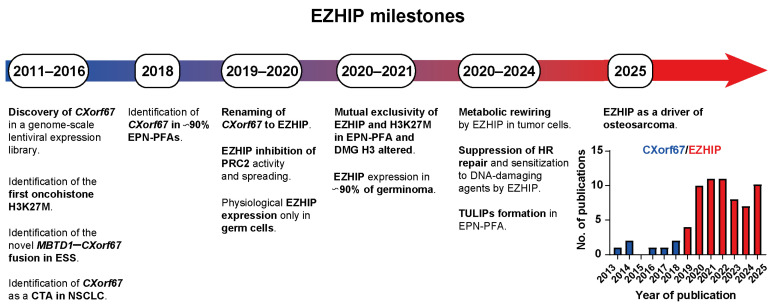
Timeline of major discoveries related to Cxorf67/EZHIP (2011–2025). Key milestones in the identification [4,19,23,24] and functional characterization of EZHIP, including its canonical role in PRC2 inhibition [10,20,21,22,25] and emerging non-canonical functions [26,27,28,29,30]. Discovery of EZHIP expression in normal tissues [18] and across cancer types is also indicated [4,31,32,33]. The landmark discovery of H3K27M is annoted for reference [5,6,7]. (Right) Bar plot showing the number of publications per year on Cxorf67/EZHIP based on a PubMed search performed using the terms “CXorf67” or “EZHIP” (last accessed: 15 October 2025), highlighting that EZHIP remains an enigmatic and underexplored field of oncology research. Abbreviations: cancer testis antigen (CTA), endometrial stroma sarcoma (ESS), homologous recombination (HR), non-small cell lung cancer (NSCLC), TULIP (type B ultra long-range interactions in PFA).

The *EZHIP* gene was first identified in the early 2010s, initially annotated as *Chromosome X open reading frame 67* (*CXorf67*), encoding a yet uncharacterized protein [19]. Its highly restricted expression during embryonic development and in adult tissues likely accounts for its late characterization in the early 2020s, when it was renamed *CATACOMB* and then *EZHIP* (the official name) following the discovery of its inhibitory function of EZH2 [20,21,22] (Figure 1).

Human *EZHIP* is an intronless gene (IG) about 1896 base pairs long, located at the Xp11.22 locus and fully embedded within introns 1–2 of the noncoding gene *RP11-348F1.2*, suggesting that it may have originated via retroposition [23,34]. Although IGs represent only ~3% of the human genome, their critical roles in cell proliferation and development, along with their restricted expression in the nervous system and testis, link them to developmental disorders and cancer [35]. IGs have been connected to cancer testis antigens (CTAs)—a large family of tumor-associated proteins normally expressed only in the testis and placenta but aberrantly expressed in diverse cancers [36]. *EZHIP* recapitulates the features of both IGs and CTAs, and was recently identified as a novel CTA in non-small cell lung cancer (NSCLC) [24]. Like many CTA genes, *EZHIP* harbors a large CpG island, which is methylated in normal somatic cells and also in nearly all cancer tissues and cell lines—potentially explaining its spatially and temporally restricted transcription [20].

**Figure 2:**
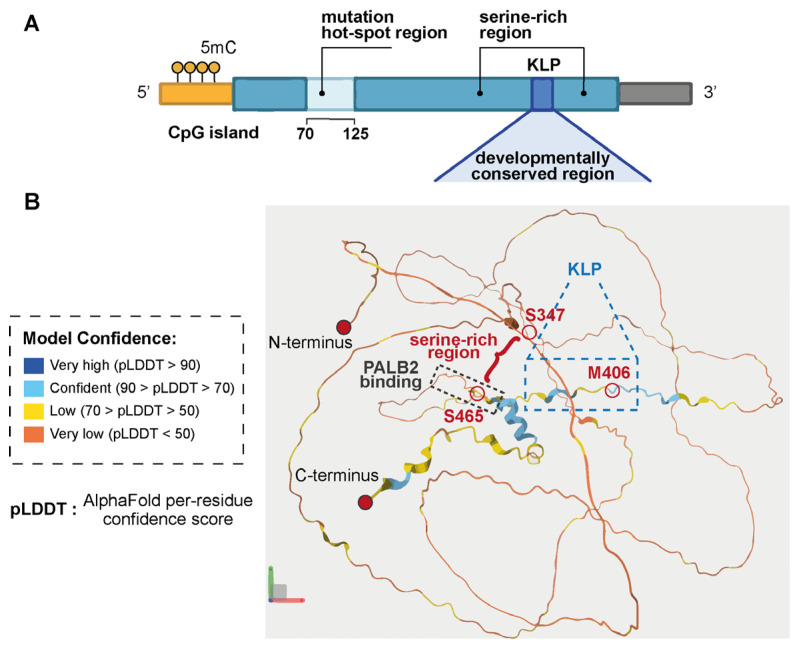
EZHIP gene and protein. (**A**) Schematic representation of the intronless, monoexonic *EZHIP* gene, harboring a large methylated CpG island (yellow), a hotspot region between codons 70 and 125 (light blue), and a conserved C-terminal region encoding a serine-rich domain (blue) that includes the short K27M-like peptide (KLP, dark blue) motif. (**B**) Predicted in silico 3D structure of the intrinsically disordered EZHIP protein (UniProt Q86X51) from www.uniprot.org. The serine-rich region, KLP motif, and partner and localizer of BRCA2 (PALB2) binding site are highlighted. Residue M406 as well as residues S347 and S465 flanking the serine-rich region are circled in red. Model confidence is indicated by color-coded pLDDT scores generated by AlphaFold.

*EZHIP* is a eutherian-specific gene that exhibits rapid evolutionary divergence across phylogeny at both the nucleotide and protein levels, in stark contrast to PRC2 complex components, including EZH2 [18]. A notable exception is a 13-amino acid sequence at the C-terminus, which is involved in modulating PRC2 activity and is highly conserved across all identified orthologs [20,21,37] (Figure 2A).

Genomic database analyses have revealed missense single nucleotide variants (SNVs) throughout the entire *EZHIP* gene and across a variety of tumors: 5.8% of endometrial carcinomas, 2.7% of melanomas, 1.4% of adenocarcinomas, and less than 1% of adult low-grade gliomas, glioblastomas, and carcinomas (Figure S1A) [4]. A notable exception is EPN-PFA, where recurrent missense mutations are detected in nearly 10% of cases, predominantly within a hotspot region between codons 70 and 125. The functional impact of these mutations is currently unknown, as they do not affect EZHIP expression, do not impair PRC2 inhibition, and show no correlation with clinico-pathological features [18,22]. Across other pediatric brain tumors, *EZHIP* mutations are exceedingly rare (~0.8%) and occur sporadically in cases of pediatric medulloblastoma (MB) and high-grade glioma (HGG), including H3K27M-positive DMG [38].

As a monoexonic gene, *EZHIP* produces a single transcript of 1512 bps, encoding a 51.9kD protein of 503 aminoacids with nuclear localization [4,18]. In adult bulk tissues, mRNA expression is highest in the testis, followed by progressively lower levels in the cerebellum, pituitary, and ovaries, with negligible expression elsewhere (Figure S1B) [39]. Interestingly, this expression profile is distinct from that of its target EZH2, which is expressed ubiquitously in both developing and adult tissues. At the protein level, EZHIP is detected in spermatogonia and round spermatids in testis, in primordial follicles and oocytes in ovaries, and in the placenta.

During human embryonic development, *EZHIP* expression is largely unknown. It is highly transcribed in primordial male and female germ cells (GCs) from week 5 of gestation. While in male gonads the expression sharply declines by 16 post-conceptional weeks, only to rise again during adolescence, female gonads maintain high *EZHIP* expression up to 18 weeks, the latest point assessed [40]. Notably, *EZHIP* is absent from embryonic stem cells (ESCs) and somatic cells [18,39,41], showing minimal expression—if any—in the developing brain, with only a transient peak in the choroid plexus around week 10 [40,42]. Concordantly, single-cell RNA-seq datasets from first- and second-trimester human brain samples show no detectable *EZHIP* expression in any brain cell type [38,43,44]. Although current human developmental atlases remain incomplete, these findings do not support the hypothesis that the temporal window for the cell of origin of EZHIP-driven tumors overlaps with germ cell formation [45].

Overall, this expression pattern is consistent with *EZHIP* functioning as a “germline-reprogramming-responsive” gene, implicated in epigenetic reprogramming by the selective removal of H3K27me3 from the promoters of canonical PRC2 targets that occurs post-fertilization [18,46].

### 2.2. EZHIP: Structure and PRC2 Inhibitory Function

Currently, no X-ray crystallographic structure of EZHIP is available. The in silico 3D model (UniProt Q86X51) indicates that EZHIP is an intrinsically disordered protein (IDP) lacking any known protein domain (Figure 2B) [47]. However, this model exhibits low per-residue confidence, suggesting structural uncertainty. An N-terminal region between residues 70 and 125—the one enriched in SNVs in EPN-PFA—stands out as less disordered and may contain protein–protein interaction domain(s), implying potential functional significance [4]. At the C-terminus, the protein encompasses a serine-rich sequence of 120 amino acids, which includes the highly conserved 13-amino acid motif (residues 404–416). This motif, referred to as the K27M-like peptide (KLP), closely resembles the 23–31-amino acid sequence of H3 harboring the K27M mutation, which aligns with M406 of EZHIP [21]. Three independent groups have demonstrated that EZHIP in EPN-PFA functionally mimics the H3K27M oncohistone in H3K27-altered DMG to cause global H3K27me3 reduction [20,21,22]. Mechanistically, EZHIP KLP binds to EZH2 and interferes with its lysine methyltransferase activity in a manner remarkably similar to the corresponding sequence in H3K27M. The EZHIP consensus motif even more closely aligns with the computationally determined “optimal” EZH2 substrate sequence, potentially explaining EZHIP’ stronger inhibitory effect (IC_50_ = 4.1 μM) on PRC2 activity compared to that of H3K27M (IC_50_ = 27.87 μM) [22].

PRC2 is a chromatin regulatory complex that, in mammalian cells, dynamically regulates the spatio-temporal expression pattern of target genes during development (expanded reviews [48,49,50,51,52]). Deregulated activity plays a pleiotropic role in cancer by modulating key cellular programs, including the proliferation–differentiation balance, metabolism, and the immune response. Notably, the mechanisms underlying pediatric hindbrain tumorigenesis, although different, all converge on PRC2 function and targets during restricted developmental windows [13,53].

**Figure 3:**
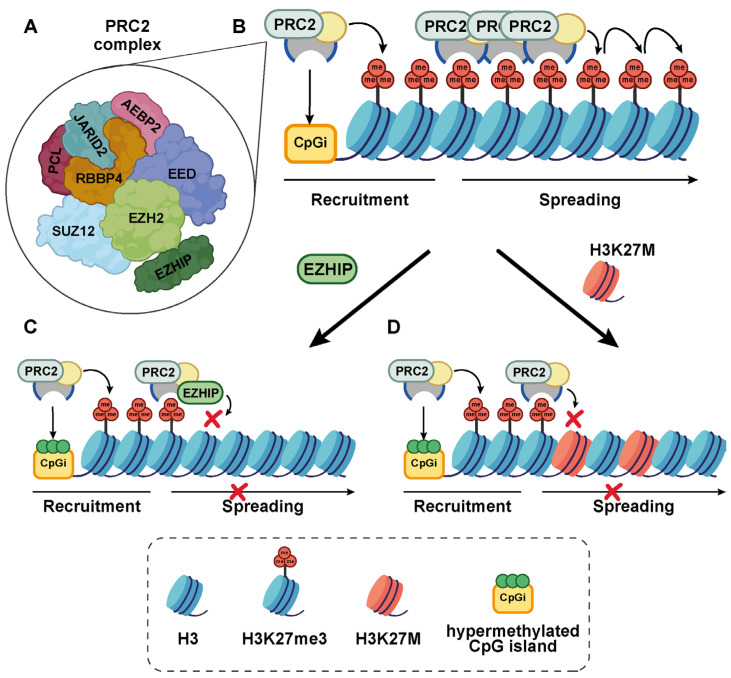
Mechanism of PRC2 methyltransferase inhibition. (**A**) Schematic representation of the PRC2 complex, composed of the core subunits EZH2, EED, SUZ12, and RBBP4, along with the accessory polypeptides AEBP2 and JARID2. Interaction between the methyltransferase EZH2 and EZHIP is indicated. (**B**) In normal cells, PRC2 is recruited to unmethylated CpG islands (CpGis), where it catalyzes H3K27 trimethylation and spreads genome-wide the H3K27me3 mark. (**C**,**D**) In EZHIP- or H3K27M-driven tumors, PRC2 is recruited to methylated CpG islands, but its spreading activity is inhibited by the competitive binding with either EZHIP (panel (**C**)) or H3K27M (panel (**D**)), leading to global loss of H3K27me3. Abbreviations: CpG island (CpGi).

The core PRC2 complex consists of EZH1/2, SUZ12, Polycomb protein embryonic ectoderm development (EED), and RBP-7/4. Additional polypeptides—AEBP2, Polycomb-like proteins (PCLs), and JARID2—allosterically potentiate PRC2 catalytic activity (Figure 3A). PRC2 is recruited to high affinity, cell type-specific nucleation sites, including unmethylated CpG islands, via its auxiliary subunits, PCLs, and JARID2, where it catalyzes high levels of H3K27me3. The H3K27me3 mark then interacts with the EED subunit, stimulating the EZH2 SET catalytic domain via allosteric activation [48,49]. This “read–write” mechanism enables PRC2 to propagate H3K27me3 into the surrounding intergenic regions, establishing broad repressive domains (Figure 3B).

EZHIP does not prevent PRC2 recruitment to chromatin. Rather, it associates with chromatin via PRC2, as a knockout of the H3K27me3 binder EED abolishes the EZHIP ChIP signal [10]. Several mutagenesis studies have identified M406 of EZHIP as a critical residue for inhibiting global H3K27me3 [20,22,37]. M406 likely interacts with the active site of EZH2 via van der Waals forces, while the adjacent arginine 405 (R405) anchors the complex via salt bridges with EZH2 residues aspartic 652 (D652) and glutamine 648 (Q648) [10]. The EZHIP–PRC2 interaction is facilitated by S-adenosyl methionine (SAM), the methyl donor essential for EZH2-catalyzed histone trimethylation. EZHIP’s inhibitory potential—like that of H3K27M—is significantly enhanced when PRC2 is allosterically activated, particularly at CpG islands containing H3K27me3. There, EZHIP stabilizes a high affinity ternary complex (H3K27me3–PRC2–EZHIP) which prevents PRC2 spreading and leads to widespread loss of intergenic H3K27me3 [10,22,25] (Figure 3B,C). However, PRC2 catalytic activity is preserved at certain nucleation sites, where hundreds of CpG islands retain residual H3K27me3. H3K27me3 ChIP-seq profiling performed in EPN-PFAs and H3K27M mutant DMGs has identified approximately 500 genes—including *CDKN2A*, *CRAPB1*, and members of the *HOXA* gene family, that retain their repressive histone marks [22,37]. Of interest, *CRABP1* is highly expressed in the progenitor cells of both tumor types, but not in other brain tumors, further supporting the presence of shared molecular programs involved in human hindbrain development [12,54].

#### EZHIP Interactome: What Is Known

**Figure 4:**
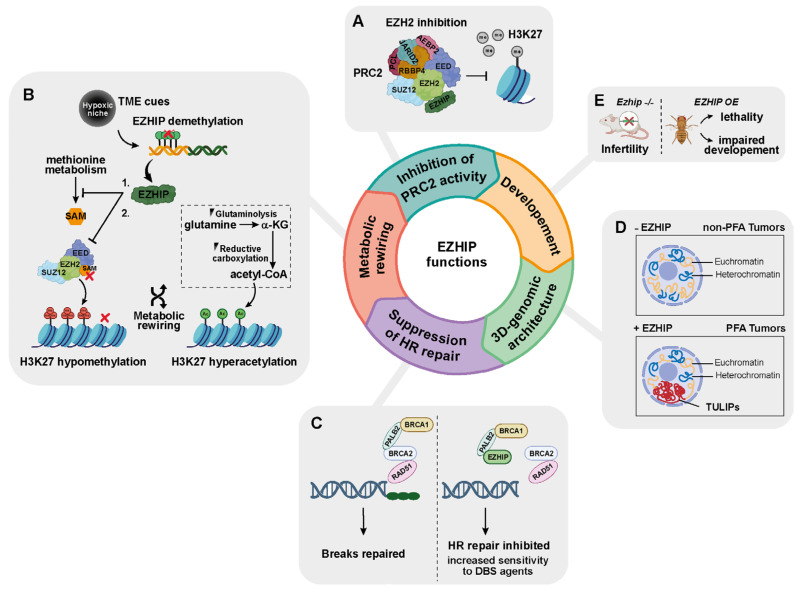
Functional roles of EZHIP in cancer (A–D) and development (E). (**A**) EZHIP inhibits PRC2 activity leading to global H3K27me3 loss. (**B**) EZHIP expression is increased by hypoxic conditions and potentially other tumor microenvironment (TME) cues and modulates metabolic pathways. Inhibition of methionine metabolism and increasing of glutaminolysis and reductive carboxylation contribute to metabolic rewiring, resulting in H3K27 hypomethylation and parallel hyperacetylation. (**C**) EZHIP interferes with homologous recombination (HR) repair by binding PALB2, which leads to impaired double-strand break (DBS) repair and increased sensitivity to DNA damaging agents. (**D**) EZHIP reshapes 3D genomic architecture in EPN-PFAs leading to TULIPs formation. (**E**) Developmental consequences of EZHIP expression in animal models: in female mice, *Ezhip* loss causes infertility, while in *Drosophila melanogaster*, *EZHIP* overexpression (OE) causes lethality and morphological abnormalities.

The identification of EZHIP as an EZH2 inhibitor has led to the discovery of additional EZHIP interaction partners, as EZHIP itself lacks enzymatic activity (Figure 4A). Co-immunoprecipitation and mass spectrometry analyses have shown strong association with PRC2.2 core components (EZH2, SUZ12, EED, RBBP4) and auxiliary proteins from both PRC2.2 (JARID2, AEBP2) and PRC2.1 (EPOP, PALI1, PHF1, MTF2, PHF19) [4,20,21,22]. The PRC2 paralog EZH1 also interacts with EZHIP, albeit with lower affinity.

Independently of its canonical role in chromatin regulation, EZHIP also interacts with several DNA repair proteins [28]. Among them, its primary binding partner is PALB2, a key mediator of homologous recombination (HR)-mediated DNA repair. EZHIP contains a short 13-amino acid motif (residues 420–432) that closely mimics the PALB2-binding region of BRCA2, allowing direct engagement of the PALB2 WD40 domain. The PALB2–EZHIP interaction competitively prevents PALB2–BRCA2 complex assembly, thereby blocking BRCA2 recruitment to DNA damage sites and ultimately impairing HR-mediated repair [28,29].

EZHIP’s intrinsically disordered structure supports multiple protein–protein interaction interfaces: the N-terminus binds RBBP4, the C-terminus engages SUZ12, EZH2, and PALB2, while interactions with JARID2 and AEBP2 likely involve the EZH2 SET domain [20]. However, EZHIP interactome may involve other binding partners—even in the cytosol—as suggested by high-throughput experiments [55], warranting further studies to clarify its impact on chromatin regulation and potentially other cellular processes (Figure S2).

## 3. The Epigenetic Landscape of EZHIP and Beyond

### 3.1. EZHIP as an Epigenetic Modulator: Evidence from Cell-Based Studies

Similarly to H3K27M mutation [56], EZHIP reshapes the epigenetic landscape via a conserved molecular mechanism that acts independently of the cellular context, from *Drosophila melanogaster* to human tumors, despite its evolutionary restriction to placental mammals [10,37] (Figure 4A). Gain-of-function experiments in various model systems lead to global H3K27me2/3 loss and focal retention at CpG islands, whereas increased H3 K27 acetylation (H3K27ac) has inconsistently been reported [26,57] (Table 1). Conversely, CRISPR–Cas9-mediated loss-of-function reverses these chromatin states. However, EZHIP contribution to epigenomic changes other than H3K27 hypomethylation remains unclear [13,16].Unlike EED loss, which destabilizes PRC2 components without affecting H3K27me3 deposition [16], EZHIP inhibits PRC2 without altering its expression or structural integrity [10,17,37]. These findings support a “Goldilocks” model in which EZHIP finetunes, rather than abolishes, PRC2 activity—a balance likely critical for tumor maintenance—highlighting PRC2 as a therapeutic vulnerability in EZHIP-expressing tumors.

Despite the fact that mechanistic insights are advancing, the phenotypic and molecular consequences of EZHIP-driven chromatin dysregulation remain to be fully elucidated. *EZHIP* consistently confers a proliferative advantage across multiple models (Table 1), although these effects are less pronounced than those observed with *EZH2* knockout (KO). In agreement, CRISPR screens in primary EPN-PFA cells identified *EZH2* and other PRC2 components—but not *EZHIP*—as essential for tumor cell growth [20]. More recently, EZHIP has been proven as an oncogenic driver in various osteosarcoma (OS) models that promotes tumor formation, maintenance, and aggressiveness, while also altering the fate commitment of mesenchymal progenitors [33].

EZHIP induces overall modest and context-dependent changes in global gene expression. In OS cells, it primarily affects the genes involved in chromatin organization, nucleosome assembly, and mesenchymal lineage specification [18,33]. In human neural stem cells (NSCs) and HEK 293 cells, *EZHIP* overexpression modulates genes linked to metabolism, neurodevelopment, and differentiation [21,26,38]. At the proteomic and metabolic levels, transgenic *EZHIP* expression in NSCs enhances glycolysis and tricarboxylic acid (TCA) cycle pathways, while suppressing polyamine metabolism.

Beyond cancer, emerging research has identified a relevant role for EZHIP in developmental epigenetics. In mouse embryonic stem cells (ESCs), *EZHIP* regulates the interplay between repressive 5-methylcytosine (5mC) and H3K27me3 [59], which in mammals is typically mutually exclusive at CpG island-rich regions. During differentiation, as ESCs exit the naïve pluripotent state, global 5mC levels increase, with a concomitant restriction of H3K27me3 to 5mC-free, CpG island-rich domains. Although 5mC accumulation is thought to antagonize H3K27me3 deposition across the genome during differentiation, recent findings suggest that aberrant *EZHIP* expression can similarly restrict H3K27me3 distribution through a 5mC-independent mechanism. Moreover, 5mC can promote the activation of certain genes—a non-canonical function that may be relevant in both early development and cancer, where dynamic switching between 5mC and H3K27me3 frequently occurs.

### 3.2. EZHIP Connects Metabolism to Epigenetic Rewiring of Tumor Cells

The impact of cellular metabolism on chromatin dynamics and epigenetics is increasingly recognized as an oncogenic driver of pediatric hindbrain tumorigenesis [60]. The growth of EPN-PFA and patient-derived cells—but not of fetal NSCs or supratentorial ependymoma—is maintained under hypoxia, which restricts the availability of specific metabolites, resulting in H3K27 hypomethylation and acetylation [27]. Mechanistically, this epigenetic shift is linked to an increased expression of EZHIP—but not of core PRC2 complex genes—alongside decreased levels of SAM [27] (Figure 4B). In human neural progenitor cells (hNPCs), *EZHIP* overexpression significantly reduces the levels of methionine and of its downstream product SAM [38]. Collectively, these findings suggest that, under oxygen deprivation and potentially other tumor microenvironmental cues, EZHIP reduces H3K27me3 both directly by inhibiting EZH2, and indirectly, by repressing methionine metabolism. Concomitantly, hypoxia-induced increase in acetyl-CoA and α-ketoglutarate (aKG) supports H3K27 hyperacetylation and activation of KDM6A/B demethylases, further contributing to H3K27me3 depletion. The hypoxic environment may thus co-opt EZHIP expression—likely through the loss of the promoter 5mC—leading to an epigenetic reshaping of gliogenic progenitor programs in the oxygen-deprived hindbrain, thereby unlocking their unrestricted proliferative potential [13,27,53,61].

In line with a metabolic rewiring in the context of H3K27me3 depletion, both EPN-PFA and H3K27-altered DMG exhibit enhanced glycolysis, TCA cycle metabolism, and glutaminolysis [26,27,62]. In EPN-PFA, this phenotype is driven by EZHIP-mediated H3K27ac enrichment at key metabolic genes, including *HK2*, *PDHA1*, and *PRKAA2*. Interestingly, the AMPK activator metformin reduces EZHIP levels while increasing H3K27me3, suppressing TCA metabolism, and inhibiting the growth of EPN-PFA cells both in vitro and in mouse models [26].

Glutaminolysis is an essential metabolic pathway that cancer cells adopt to maintain high levels of α-KG that fuel rapid proliferation. Indeed, glutaminolysis inhibitors suppress EPN-PFA and DMG H3K27M cell growth, an effect that can be reversed by exogenous α-KG [26,62]. Interestingly, in vivo magnetic resonance spectroscopy reveals high concentrations of glutamate and citrate in EPN-PFA and DMG H3K27M patients, when compared to EPN-PFB and H3-WT DMG patients, respectively. Whether EZHIP plays a role also in reshaping the metabolic profile of EZHIP-driven H3-WT DMG remains yet unexplored. The identification of metabolic vulnerabilities in tumor-specific contexts is fundamental to epigenetically rewire oncogenic pathways in EZHIP-driven EPN-PFA and, possibly, other malignancies.

### 3.3. EZHIP Is a DNA Damage Response Protein

EZHIP-mediated suppression of HR repair sensitizes EZHIP-overexpressing cancer cells to DNA double-strand break (DSB)-inducing agents, including radiotherapy and DNA-damaging chemotherapeutics, such as etoposide, doxorubicin, and platinum-based drugs [28]. Because HR-deficient cells rely on PARP-mediated DNA repair, they exhibit increased sensitivity to PARP inhibitors (Figure 4C). In line with this, preclinical in vitro and in vivo studies have demonstrated that EZHIP-expressing cells are exquisitely sensitive to PARP inhibitors, especially in combination with radiotherapy [28] or platinum-based chemotherapy [29]. Interestingly, H3K27M mutation also impairs HR repair, sensitizing cells to similar therapeutic combinations [58]. Whether PARP inhibitors could serve as a synthetic lethal strategy in tumors driven by EZHIP overexpression or H3K27M mutations warrants further investigation.

### 3.4. EZHIP Is a Modeler of 3D Chromatin Architecture

Recent lines of evidence implicate EZHIP in the organization of recurrent 3D genome structures called TULIPs (type B ultra long-range interactions in PFA), that are uniquely found in EPN-PFA among CNS malignancies (Figure 4D) [30]. These structures represent compartments of densely compacted, transcriptionally repressed heterochromatin, in contrast to Type A compartments, which correspond to loosely packed, transcriptionally active euchromatin [63]. Heterochromatin is further categorized into constitutive (H3K9me2/3-marked, gene-poor, transcriptionally inert) and facultative (H3K27me3-marked, dynamically repressed) forms. Unlike constitutive heterochromatin, facultative heterochromatin remains accessible to transcriptional machinery, making the H3K27me3-marked genomic regions transcriptionally inactive yet poised for activation. TULIP interactions might arise from the large-scale reorganization of H3K9me3-enriched chromatin, mediated at least in part by EZHIP, because transgenic *EZHIP* overexpression in hNPCs promotes the formation of large H3K9me3-positive foci, consistent with TULIP generation.

However, the exact role of TULIPs in EPN-PFA tumorigenesis remains elusive, as they do not appear to directly regulate gene expression. They may instead be involved in other DNA-related processes, such as repair, mutation rates, and replication timing. The absence of TULIPs in DMG, which also exhibits H3K27me3 depletion, is also puzzling.

### 3.5. EZHIP and Animal Models

Compared to PRC2 components, whose deletion in mice is embryonically lethal [64,65], EZHIP appears to play a relatively modest role during mammalian development (Figure 4E). *EZHIP* KO mice show minimal consequences, including loss of *EZHIP* expression in GCs, accompanied by a global increase in H3K27me2/3 deposition, without affecting somatic cells or altering other epigenetic marks [18]. This histone modification shift is accompanied by modest changes in the transcriptome of GCs, with only about 100 genes showing differential expression. Moreover, adults display no overt developmental abnormalities and are phenotypically undistinguishable from their wild-type counterparts. *EZHIP* −/Y males display normal spermatogenesis, GC populations, sperm motility, and fertility, whereas homozygous KO females develop smaller ovaries compared to heterozygous (+/−) and wild-type counterparts, along with a marked, age-dependent infertility.

A recent work has demonstrated that EZHIP has dramatic developmental consequences in *Drosophila melanogaster* [66]. The ubiquitous transgenic expression of either *EZHIP* or *H3K27M* is lethal, though *EZHIP* exerts a more potent developmental impact. Indeed, *EZHIP* expression causes death at the third-instar larval stage, whereas *H3K27M* expression allows progression to pupation. Similarly, while the tissue-specific expression of *H3K27M* impairs wing formation, that of *EZHIP* completely abolishes it. These distinct developmental outcomes reflect the relative biochemical potency of both PRC2 inhibitors and indicate that cell context and/or additional factors modulate their activity (Figure 4E).

No in vivo model of EZHIP-driven tumorigenesis currently exists, suggesting that EZHIP is necessary, but not sufficient, to initiate tumor formation, with additional oncogenic hit(s) being required. Transgenic *EZHIP* expression in *NES*- or *GFAP*-expressing neonatal hindbrain progenitors promotes tumorigenesis only when combined with *PDGFA* overexpression and *TP53* loss [67]. The M406K point mutation significantly prolongs survival, but still retains oncogenic potential, which suggests the involvement of additional interactor(s) or mechanisms in *EZHIP* tumor-promoting activity. These findings parallel those from DMG mouse models, in which the expression of the *H3K27M* transgene in ESCs [68] or NSCs [69,70] drives de novo gliomagenesis only in the context of *TP53* deficiency and constitutive PDGFR activation. Consistently, *EZHIP* expression on its own is not sufficient to transform mesenchymal stem cells and generate an OS model in mice [33]. Further mouse models more closely mirroring the genetic backgrounds of human EPN-PFA or H3-wild type (H3-WT) DMG are needed to fully elucidate EZHIP’s oncogenic potential and guide targeted therapies.

Table 2 summarizes the common mechanisms of PRC2 inhibition mediated by EZHIP and H3K27M, while highlighting important biological distinctions between them.

**Table 2:** Shared and distinct biological consequences of EZHIP and H3K27M.

Feature	EZHIP	H3K27M
Mode of PRC2 interaction	Non-histone PRC2 inhibitor [20,21,22]	Oncohistone incorporated into chromatin [6]
PRC2 inhibition mechanism [20,21,22]	EZH2 active-site engagement	EZH2 active-site engagement
Reversibility of PRC2 inhibition	Potentially reversible	Persistent due to chromatin incorporation
EZH2 inhibitory potency [22]	Higher (IC_50_ = 4.1 μM)	Lower (IC_50_ = 27.87 μM)
Inhibition of PRC2 spreading activity [10]	Yes	Yes
Global loss of H3K27me3 [20,21,22]	Yes	Yes
Focal gain of H3K27me3 at CpG islands [22]	Yes	Yes
Inhibition of homologous recombination	Yes [28]	No
Developmental lethality (*Drosophila*) [66]	Early larval lethality	Later developmental arrest
3D chromatin reorganization (TULIPs) [30]	Present (EPN-PFA)	Absent (DMG)

## 4. A Clinical Standpoint

### 4.1. EZHIP in Pediatric Brain Tumors

#### 4.1.1. EZHIP Is a Biomarker of EPN-PFA

EZHIP is highly expressed in EPN-PFA among all pediatric brain tumors and is now recognized as a well-validated diagnostic biomarker to distinguish PFA from PFB [31,71]. Its prognostic significance, however, remains controversial. Several studies report an inverse correlation between EZHIP abundance and both progression-free survival and overall survival [26,72], while EZHIP-negative EPN-PFAs are associated with better outcomes and more differentiated cellular populations [45,73]. Other groups, however, found no link between EZHIP expression and clinical behavior [8]. Longitudinal immunohistochemical and epigenetic analyses revealed no differences in H3K27me3 or EZHIP expression between matched primary and relapsed EZHIP-expressing EPN [74].

Immunophenotypically, EZHIP-positive PFAs show evenly diffuse nuclear staining that parallels EZH2 expression, suggesting an overexpression of inactive EZH2, similar to H3K27M-mutant gliomas [8]. Across patients, staining appears to vary by tumor location, being most abundant in the PFA of the roof and lateral recess of the fourth ventricle [26]. EZHIP immunopositivity is typically accompanied by high H3K27ac and low H3K27me3, the latter inversely correlated with CA-9 staining, a surrogate for hypoxic areas [27]. Nonetheless, EZHIP has also been observed in both normoxic and hypoxic tumor regions [26].

The fifth CNS WHO Classification integrates histology with state-of-the art molecular diagnostics, allowing the powerful unsupervised classification of pediatric brain tumors into clinically meaningful molecular subgroups [1]. A fundamental breakthrough has been genome-wide DNA methylation profiling, which unifies morphologically diverse tumors under shared molecular signatures, while reassigning diagnostically ambiguous cases [70]. In this scenario, certain DMGs have been reclassified as EPN-PFA, and vice versa, independently of their molecular hallmark.

Indeed, a subset of EZHIP-negative EPN-PFAs, confirmed by methylation profiling, harbor H3K27M mutation, accounting for 1–7% overall and up to 69% in the PFA-1f subgroup [4,8,9,75]. These tumors exhibit nearly two-fold higher EZHIP promoter methylation than their H3-WT counterparts, in accordance with the mutual exclusivity of EZHIP expression and H3K27M mutations [4,37]. Because of their rarity, little clinical literature is available. Histologically, H3K27M-mutant EPN-PFAs resemble EZHIP-positive EPN-PFAs, being well-circumscribed, high-grade tumors with pseudorosettes, rosettes, and calcifications, features rarely seen in DMGs [9]. Clinically, they do not differ significantly from EZHIP-positive PFAs in terms of overall survival or progression-free survival. However, they tend to occur in older children (median age 6 years vs. 2.4 years) and are more often located in midline structures [9,75]. Interestingly, unlike DMGs where H3.3 mutations predominate [76], approximately 85% of H3K27M-mutant PFAs harbor H3.1 mutations [9].

Despite distinct clinical, radiological, and histomolecular features, overlap exists between EPN-PFA and DMG, H3K27-altered. Rare DMGs with H3K27M mutation have shown some well-circumscribed areas with typical ependymal features (pseudorosettes and true rosettes), yet the outcomes resemble DMG rather than PFA [9]. Reciprocally, a pontine tumor with ependymal morphology and infiltrative growth, initially diagnosed as EZHIP-overexpressing DMG, have been reassigned as EPN-PFA by methylation profiling [77,78]. A similar pontine biphasic case showed one solid OLIG2-negative component with ependymal features and one infiltrative OLIG2-positive component, both presenting H3K27me3 loss and EZHIP expression [79]. DNA methylation profiling classified this case as DMG, H3 K27-altered, with ependymal features.

Overall, posterior fossa tumors with ependymal features—defined by either EZHIP expression or H3K27M mutations—exhibit a distinct DNA methylation profile shaped more by the cell of origin than by the specific driver alteration.

#### 4.1.2. EZHIP Defines a Subtype of H3K27-Altered DMG

DMGs, H3K27-altered, represent a biologically homogeneous yet molecularly heterogeneous group of neoplasms [1]. They include four molecular subtypes, encompassing alternative mechanisms of H3K27 trimethylation loss beyond H3K27 mutations: H3.3-mutant, H3.1-mutant, H3-WT with EZHIP overexpression, and EGFR-mutant DMG, the latter subgroup either overexpressing EZHIP or harboring H3K27M [45,79,80].

H3-WT DMGs account for 4–15% of cases and are characterized by near-universal EZHIP overexpression at mRNA and protein levels [31,32,81]. Despite intact H3, their methylation profiles cluster closer to the H3K27M counterparts than EPN-PFA. However, they display unique transcriptomic and methylomic signatures, including differential methylation of homeobox genes [32,82]. EZHIP overexpression in H3-WT DMGs is thought to result from promoter hypomethylation, as EZHIP is consistently unmethylated even though these tumors show more global CpG islands hypermethylation than H3K27M DMGs [82]. The most prevalent genetic alterations co-occurring with *EZHIP* involve *ACVR1*, *PIK3CA*, *TP53*, and *EGFR*, with *ACVR1* and *EGFR* mutations being mutually exclusive [81,82,83].

Clinically, H3-WT DMGs present with later onset (median age, 8 years) versus H3.1-K27M (5 years) and H3.3-K27M (6–7 years) cases [32,45]. Median overall survival is ~15 months, similar to H3.1-K27M DMG but slightly longer than H3.3-K27M (10.8 months) [32,81], although other studies report nearly overlapping outcomes [82,83].

Copy number analysis shows that EZHIP-positive and H3.1-K27M DMGs share alterations with EPN-PFA [12]. Both subtypes exhibit fewer arm-length copy number changes and focal amplifications/deletions than H3.3-mutant DMGs, indicating lower genomic instability. Gain of chromosome arm 1q occurs in ~50% of EZHIP-positive and ~40% of H3.1-K27M tumors. Interestingly, while 1q gain predicts poor outcomes in ~25% of EPN-PFA [84], it is paradoxically associated with improved overall survival in H3.1-K27M DMGs [12].

A distinct subgroup, EGFR-mutant DMGs, has also recently been defined [1], which shows a unique epigenetic profile, preferential thalamic location, and frequent EGFR gene amplification and/or mutation [85]. Global H3K27me3 loss in this subtype arises either from H3K27M mutation or EZHIP overexpression. Clinically, they carry a poor prognosis, consistent with H3K27M- and EZHIP-positive DMGs. Although this diagnostic category currently provides limited prognostic or therapeutic guidance, comprehensive molecular profiling of DMGs may eventually inform targeted therapeutic strategies.

#### 4.1.3. Germinoma

In other pediatric CNS tumors, EZHIP expression has been occasionally reported apart from germinomas, which display EZHIP positivity in most cases (94%; 29/31) at levels comparable to EPN-PFAs [4,31]. In germinoma, *EZHIP* lacks mutations, but instead exhibits promoter hypomethylation, leading expectedly to the global loss of H3K27me3. In a cohort of adult and pediatric patients (*n* = 370) with different CNS GC tumors (GCTs), EZHIP positivity was detected exclusively in the nuclei of normal germ and germinoma cells, but not in other GCT subtypes [86].

#### 4.1.4. Medulloblastoma (MB)

EZHIP immunopositivity has been rarely found in MBs, where it has been reported in only 1 of 10 WNT-activated cases in a cohort of 40 MBs of different histologies [31]. Analyses of publicly available transcriptomic datasets [87] confirmed that *EZHIP* expression is generally very low across MBs. However, microarray data from the Northcott cohort (285 pediatric MBs) [88] demonstrated significantly higher EZHIP expression in the SHH subgroup compared with group 3 (G3) and group 4 (G4) tumors, reaching levels comparable to those of *GLI1* in the same subgroup (Figure S3A,B). This finding is consistent with the strong EZHIP expression in DAOY cells, a widely used in vitro model of SHH subgroup MB.

#### 4.1.5. Atypical Teratoid/Rhabdoid Tumor (AT/RT)

Currently, only a single study has investigated *EZHIP* expression in AT/RT, reporting focal EZHIP immunopositivity in one of three analyzed cases [31].

#### 4.1.6. MYCN-Amplified Glioblastomas (MYCN_GBMs)

EZHIP overexpression has been episodically found in MYCN_GBMs, with expression detected in two of fifteen cases, (13%) and absent in non-MYCN-amplified GBMs of the same subtype [32]. However, these findings are not consistent across studies, with another cohort showing no EZHIP immunopositivity in nine examined MYCN-driven high-grade gliomas (MYCN-HGGs) [31].

### 4.2. EZHIP and Adult Cancers

A driver role for EZHIP has been firmly established only in pediatric EPN-PFAs and DMG H3-WT, whereas its contribution to adult malignancies remains largely undefined (Table 3). Functional evidence in adults is currently restricted to OS, where, however, EZHIP lacks autonomous transforming capacity, consistent with a co-driver rather than primary driver role. Similarly, in endometrial stroma sarcoma (ESS) and Merkel cell carcynoma (MCC), EZHIP oncogenic involvement as a co-driver is inferred from recurrent fusion events or co-occurrence with viral oncogenes (e.g., Merkel cell polyomavirus). In NSCLC, EZHIP appears to function predominantly as a biomarker, rather than a causative oncogenic factor.

#### 4.2.1. Endometrial Stroma Sarcoma (ESS)

The first evidence implicating *EZHIP* in oncogenesis came from the discovery of a recurrent chromosomal translocation t(X; 17) (p11.2; q21.33) in low-grade ESS [23]. The resulting fusion *MBTD1–CXorf67* encodes a chimeric protein of 839 amino acids, composed of nearly the entire *MBTD1* sequence (lacking the last 39 residues) fused to the C-terminal half of *EZHIP*, which contains the serine-rich region [20]. MBTD1 is a chromatin regulator with functions associated with Polycomb group (PcG) proteins, and genes with PcG-related roles are frequently implicated in ESS-associated rearrangements. Specifically, MBTD1 is thought to recruit PcG complexes to Polycomb response elements, thereby modulating the expression of specific tumor suppressor genes [23].

#### 4.2.2. Non-Small Cell Lung Cancer (NSCLC)

EZHIP has been identified as a novel CTA in a comprehensive discovery study of the CTA landscape in NSCLC [24]. Strong nuclear EZHIP expression occurs in 3.2 to 11.4% of cases across squamous cell carcinoma and adenocarcinoma subtypes [24,89]. Network analyses of CTA expression pattern revealed the co-expression of EZHIP with other CTAs such as *PASD1*, *MAGEA4*, *MAGEC2*, and *TKTL1*. However, no significant correlation has been established between EZHIP expression and clinical outcomes.

Within the immune microenvironment, high EZHIP levels are significantly associated with plasma cell infiltration (CD138), while low EZHIP correlates with PDL1 levels (*p* < 0.01) [89]. These findings suggest that EZHIP may influence tumor–immune interactions, which may be exploitable in immunotherapeutic approaches.

#### 4.2.3. Merkel Cell Carcinoma (MCC)

MCC is a rare but aggressive skin cancer with limited therapeutic options [90]. The integration of Merkel cell polyomavirus (MCPyV) and the expression of viral oncogenes are observed in ~80% of cases and represent major oncogenic drivers [91]. Given the low mutational burden of MCPyV-associated tumors, epigenetic dysregulation leading to the global loss of H3K27me3 occupancy has been proposed as a pathogenetic mechanism [92]. However, findings on H3K27me3 loss remain controversial. H3K27 hypomethylation is found in 80% of MCCs in one study [92], predominantly in virus-positive tumors, while being only ~25% and confined to virus-negative tumors in another study [89]. H3K27me3 loss has been associated with high EZHIP expression, secondary to promoter DNA hypomethylation [90], in approximately 16% of MCCs [91]. These observations suggest that EZHIP may contribute to MCC pathogenesis through epigenetic deregulation, although its interplay with viral status requires further clarification.

#### 4.2.4. Osteosarcoma (OS)

Aberrant EZHIP expression has very recently been reported in ~20% OS patients across two independent cohorts (total number = 83 patients). Reduced H3K27me3 deposition was observed in over 30% of samples [33]. No correlation between EZHIP and clinical variables, such as sex, age, or the presence of metastases at diagnosis or relapse, has been established. In contrast, decreased H3K27me3 levels were enriched in samples from chemo-resistant patients as well as in post-therapy and relapse samples. Histologically, EZHIP and H3K27me3 immunopositivity were highly heterogenous, with densely positive tumor areas adjacent to negative ones. At the cellular levels, EZHIP-positive nuclei consistently exhibited H3K27me3 loss, although extensive depletion was also observed independently of EZHIP expression, suggesting that multiple mechanisms may regulate H3K27me3 abundance in OS. It remains unknown whether this observation also applies to pediatric brain tumors, a question that may provide mechanistically relevant insights.

## 5. From Undruggable to Vulnerable: Strategies to Target EZHIP-Driven Tumors

EPN-PFAs and H3K27-altered DMGs are intrinsically chemoresistant tumors, in which RT remains the primary therapeutic choice whenever gross total surgical resection is unfeasible—a frequent scenario in DMGs. Given their convergent epigenetic landscapes, these tumors potentially harbor shared vulnerabilities, which could be exploited therapeutically, including their dependence on EZHIP.

**Figure 5:**
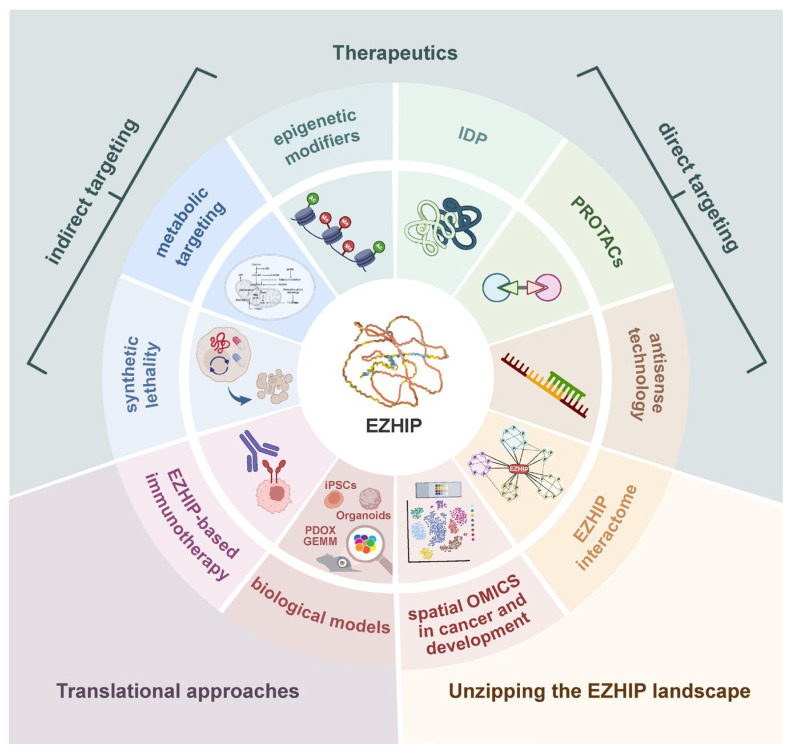
A visual overview of emerging research directions of the EZHIP in cancer. The schematic highlights potential therapeutic strategies (direct and indirect targeting), translational approaches, and the EZHIP landscape.

Although EZHIP’s highly restricted expression in normal tissues, including brain, makes it a potentially ideal candidate, its intrinsically disordered structure poses substantial challenges to direct pharmacological intervention. For this reason, current strategies mainly focus on modulating the EZHIP-driven hypomethylated/hyperacetylated epigenome rather than the protein directly. The following section outlines a summarized picture of the most promising approaches under investigation to therapeutically target the EZHIP-associated landscape (Figure 5).

### 5.1. Direct Targeting

#### 5.1.1. EZHIP as an IDP

IDPs are characterized by transient, highly dynamic conformations, that interconvert on rapid timescales. This structural plasticity allows their binding promiscuity, multifunctionality, and broad biological functions [93,94]. Consequently, the classical “sequence–structure–function” paradigm for folded proteins has been replaced by the “sequence–ensemble–function” framework for IDPs [95]. Mounting evidence highlights a key role for IDPs in cancer and neurodegenerative diseases, which has spurred growing efforts to target them therapeutically [96].

However, the development of strategies to directly inhibit IDPs like EZHIP poses challenging hurdles. The absence of stable unique structures, inherent conformational heterogeneity, and capacity for multiple protein–protein interactions collectively make traditional structure-based drug design approaches poorly suited for IDPs. Therefore, a range of alternative strategies have been developed, including protein–protein interaction inhibitors, phenotypic or high-throughput screening approaches, as well as chemoproteomic and biophysical strategies [94,96,97].

Recent advances in computational modeling and artificial intelligence (AI) have further expanded the toolkit for IDP drug discovery [98,99]. The integration of next-generation experimental methodologies with AI-driven structural prediction and compound screening holds promise for addressing previously undruggable proteins. In the specific case of EZHIP, elucidating its precise structural and chemical interactions remains particularly complex due to its functionally relevant elements distributed throughout the protein and poorly defined interactome [16].

#### 5.1.2. Proteolysis Targeting Chimeras (PROTACs)

PROTACs have emerged as a novel therapeutic modality of selectively degrading proteins that are otherwise difficult to target with conventional approaches [100]. More than 30 PROTACs are currently undergoing clinical trials and the field is now poised for its first regulatory approval in oncology [101].

PROTACs function by tethering a ligand for the target protein to an E3 ubiquitin ligase recruiter, thereby promoting ubiquitination and subsequent proteasomal degradation. This technology has been proposed as a potential strategy to tackle EZHIP [45]; however, its efficacy relies on the formation of a stable ternary complex, a challenge for IDPs like EZHIP, which lack defined ligand binding pockets or structural domains. Indeed, attempts to apply PROTACs to relevant oncogenic IDPs, including MYC [102,103] and YAP [104], have thus far been unsuccessful.

#### 5.1.3. Antisense Technologies

The vast majority of disease-related proteins are untargetable by conventional strategies, such as small-molecule drugs and antibodies. A promising alternative is represented by nucleic acid-based therapeutics, including antisense oligonucleotides (ASOs) [105,106]. ASOs hybridize with complementary RNA sequences and promote RNase H-mediate cleavage of target transcripts. With respect to traditional drugs, nucleic acid-based therapeutics offer several advantages, including applicability to virtually any therapeutic target, ease of design, and relatively low production costs.

A landmark preclinical study has recently demonstrated that antisense knockdown of H3.3K27M in DMG patient cells restores H3K27 trimethylation, inhibits gliomagenesis, promotes differentiation, and prolongs survival in mouse models [107], providing a proof-of-concept for ASO therapies in DMGs, and potentially in EZHIP-driven tumors.

### 5.2. Indirect Targeting

The indirect targeting of EZHIP-associated vulnerabilities spans diverse drug classes, including traditional and experimental cytotoxic agents [108,109,110], targeted therapies [111], epigenetic modifiers [112], and immunotherapy [113].

A paradigmatic example involves targeting the oncosuppressor CDKN2A pathway, which is consistently epigenetically silenced by H3K27 trimethylation, leading to unchecked CDK4/6 activation and tumor cell cycle progression. Hence, CDK4/6 inhibitors have gained interest as a therapeutic strategy against pediatric brain tumors [114], with multiple clinical trials assessing their safety and efficacy as monotherapies or combination regimens (https://clinicaltrials.gov/, accessed on 30 september 2025).

Another target under active clinical investigation is EZH2 [115,116]. However, the dual role of EZH2 in EPN-PFAs and DMGs—acting both as a tumor suppressor and an oncogene [52]—may underlie the inconsistent results across studies in vitro and in vivo, with either growth-inhibitory [115,117] or null effects having been reported [8,118].

Histone deacetylase inhibitors have been extensively studied in preclinical models and are now informing early-phase clinical trials. However, the epigenetic landscape of EPN-PFAs and H3-WT DMGs is not only marked by H3K27me3 loss but also by a reciprocal gain in histone acetylation [2,7,119]. Marks such as H3K9ac and H3K27ac activate transcription at neurodevelopmental enhancers and super-enhancers, driving aberrant tumor cell differentiation [12,17,57]. For example, in DMGs, the oncogene MYC is highly expressed due to strong enrichment at H3.3K27M- and H3K27ac-associated super-enhancers [120]. Histone acetyltransferase inhibitors represent a promising, though still underdeveloped, therapeutic class for countering pervasive transcription in these tumors. Nonetheless, significant technical hurdles remain, particularly regarding specificity, efficacy, and CNS delivery [121,122].

Metabolic dysregulation has also emerged as a vulnerability in EZHIP-associated tumors, opening new therapeutic avenues. A compelling target is SAM, a critical cofactor sustaining the altered epigenetic landscape of EZHIP-expressing tumors by supporting EZHIP–KLP–PRC2 complex formation. In this context, the inhibition of methionine adenosyltransferase 2A (MAT2A)—the enzyme responsible for SAM biosynthesis—has been proposed as a novel yet unexplored therapeutic strategy [123]. However, given SAM’s essential role in PRC2 function, its systemic depletion could have unintended paradoxical effects.

### 5.3. EZHIP-Based Immunotherapy

The immune microenvironment of EZHIP-expressing PFAs and DMGs remains largely unknown, although pediatric brain tumors are typically “cold”, with low mutational burden, weak tumor antigen presentation, limited immune cell infiltration, and an intact blood–brain barrier [124,125,126]. As highly immunogenic neoantigens, CTAs represent attractive targets for CTA-guided immunotherapies. In lung cancer, EZHIP-expressing tumor cells are associated with CD138+ plasma cell infiltration [24]. Despite its potential role as a CTA, EZHIP is not suitable for CAR T cell therapy because of its nuclear localization. However, T cells expressing an engineered T cell receptor (TCR-T cells) are emerging as a promising alternative approach to target intracellular, non-membrane proteins [127].

Alternatively, EZHIP-associated antigens could represent an immune-targetable feature of EZHIP-driven tumors. For example, EZHIP overexpression induces IL13RA2 in HEK293T cells [21]. Interestingly, in a genetically engineered mouse model, IL13RA2 expression conferred a significant response to CAR T cell therapy, resulting in prolonged survival (46 days vs. 28 days in controls; *p* < 0.0001) and 25% long-term survival [128]. However, although CAR T cell therapy is a breakthrough treatment, the associated side effects—including severe inflammatory responses and neurotoxicity—pose significant challenges, especially in children with brain tumors [129]. Careful optimization will be required for safe clinical translation.

### 5.4. Intratumoral EZHIP Heterogeneity: Implications for Therapeutic Development

An important consideration for EZHIP-informed therapeutic strategies is the growing evidence of the intratumoral heterogeneity of EZHIP expression, particularly evident in EPN-PFAs and in OS. The coexistence of EZHIP-positive and -negative tumor compartments, together with the EZHIP-independent mechanisms of H3K27me3 depletion, suggests that EZHIP expression alone does not fully capture the functional epigenetic landscape of these tumors. This has direct implications for therapeutic targeting.

For example, for antisense-based strategies aimed at EZHIP suppression, heterogeneous expression raises the possibility of incomplete target engagement and the selective survival of EZHIP-negative or low-expressing subclones that retain a tumor-propagating capacity. Similarly, heterogenous EZHIP-driven loss of H3K27me3 may confer distinct metabolic dependencies, including altered hypoxia tolerance or α-ketoglutarate-sensitive chromatin regulation, which may not be shared by adjacent EZHIP-negative cells. Moreover, because EZHIP impairs DNA repair, only a subset of tumor cells may be sensitized to PARP inhibition coupled with DNA damage–response–targeting approaches.

Future development of EZHIP-directed therapies will likely require biomarker frameworks and rational combination strategies that target both EZHIP-dependent and -independent vulnerabilities to overcome intratumoral epigenetic heterogeneity and prevent adaptive resistance.

## 6. Current Gaps and Future Directions

Despite the increasing recognition of EZHIP as a driver of PRC2 dysfunction in cancer, its developmental role, tumor-specific regulation, and mechanistic contributions remain incompletely defined.

Current evidence supports a model in which EZHIP is not a core developmental regulator, but rather a highly context-dependent modulator of PRC2 activity, whose physiological function is largely confined to the germline. Loss-of-function studies in mice indicate that EZHIP is largely dispensable for embryonic viability and gross somatic development [18], arguing against an essential role during early embryogenesis. However, the observation of age-dependent female infertility in *EZHIP*-deficient mice suggests that EZHIP is not fully redundant. Instead, it likely contributes to the finetuning of Polycomb-mediated repression during periods of heightened epigenetic plasticity, such as germ cell maturation and epigenetic reprogramming. Whether EZHIP plays similarly subtle or transient roles during early embryogenesis or neural lineage specification remains unknown.

The existing knowledge of its developmental expression relies largely on bulk tissue analyses, which may obscure transient or cell type-specific roles that escape detection by current transcriptomic and epigenomic atlases. Similar limitations complicate interpretation in EZHIP-positive tumors, where low global mRNA levels contrast with strikingly heterogeneous protein expression. Whether such heterogeneity is sufficient to drive tumor-wide epigenetic effects, as observed for H3K27M, remains unresolved.

Mechanistically, EZHIP reactivation in cancer has been linked to the hypomethylation of its large CpG island [20,59], although the upstream drivers of this epigenetic state remain unclear. Its selective expression in certain brain tumors, germinomas, and osteosarcomas, often in cooperation with specific genetic backgrounds, supports a context-dependent mechanism of action. Gain-of-function models demonstrate that ectopic EZHIP expression can profoundly disrupt development, reinforcing the concept that the misexpression rather than the loss of EZHIP underlies the pathology. This framework reconciles the apparent paradox between EZHIP’s limited physiological role and its potent oncogenic activity, positioning it as a developmentally constrained epigenetic modulator whose misexpression—rather than loss—is the primary driver of pathology.

## 7. Conclusions

EZHIP exemplifies how a germline-restricted, evolutionarily recent gene can be co-opted in cancer to drive epigenetic dysregulation. Its aberrant reactivation creates exploitable vulnerabilities, supporting therapeutic strategies that target downstream consequences rather than EZHIP itself. The continued integration of functional models and context-resolved epigenomic approaches combined with innovative therapeutic strategies—including antisense technologies and immunotherapy—will be essential to translate EZHIP from a diagnostic marker into an actionable target in pediatric neuro-oncology.

## Figures and Tables

**Table 1:** Summary of EZHIP-dependent epigenetic and functional alterations across cells.

Cell Line	*EZHIP* Status	H3K27me3	H3K27ac	H3K27me3 Genome Distribution	OMICS	Functional Outcomes
EZHIP-expressing cells						
DAOY (MB) ^1^ [4]	KO ^8^	↑	↓			proliferation ↓
U2OS (OS) ^2^ [18,33]	KO	↑	↓	spreading from CpGi ^10^	500 genes (most down)	tumorigenicity ↑ SC ^11^-like phenotype
mGCs ^3^ [18]	KO	↓	=		125 genes (most down)	
EZHIP-negative cells						
MG63 (OS) [33]	OE ^9^	↓		restricted at CpGi		tumorigenicity ↑ SC-like phenotype
KOSH (OS) [33]	OE	↓		restricted at CpGi		tumorigenicity ↑ SC-like phenotype
MSCs ^4^ [33]	OE	↓				proliferation ↑ no tumorigenicity altered differentiation
HEK293T [21,22,58]	OE	↓	↑, =		200 genes (most up)	
hNSCs ^5^ [28,30,38]	OE	↓	↑		2800 genes metabolomics	proliferation ↑
mNSCs ^6^ [26]	OE	↑	↑		2400 proteins	proliferation ↑ differentiation ↓
MEFs ^7^ [22]	OE	↓		restricted at CpGi	500 genes (most up)	
S2 cells (*D. melanogaster)* [10]	OE	↓				

^1^ medulloblastoma (MB); ^2^ osteosarcoma (OS); ^3^ mouse germ cells (mGCs); ^4^ mesenchimal stem cells (MSCs); ^5^ human neural stem cells (hNSCs); ^6^ mouse neural stem cells (mNSCs); ^7^ mouse embryonic fibroblasts (MEFs); ^8^ knockout (KO); ^9^ overexpression (OE); ^10^ CpG island (CpGi); ^11^ stem cell (SC); increase (↑); decrease (↓); = no change.

**Table 3:** EZHIP expression and inferred functional roles in adult malignancies.

Tumor Type	Expression (%)	EZHIP Role	Functions
ESS	5%	Co-driver	- *MBTD1–CXorf67* recurrent fusion - modulation of PcG function
NSCLC	3–11%	Biomarker	- co-expressed with other CTAs - potential role in tumor–immune interactions - no clear prognostic link
MCC (adult)	16%	Co-driver	- secondary to viral oncogenes - associated with H3K27me3 loss
OS	20%	Co-driver/biomarker	- no clear link to clinical outcomes - associated with H3K27me3 loss

## Data Availability

No new data were created or analyzed in this study. Data sharing is not applicable to this article.

## Supplementary Materials

The following supporting information can be downloaded at https://www.mdpi.com/article/10.3390/ijms27020963/s1.

## Abbreviations

The following abbreviations are used in this manuscript:

5mC	5-methylcytosine
*a*-KG	Alpha-ketoglutarate
ASOs	Antisense oligonucleotides
AT/RT	Atypical teratoid/Rhabdoid tumor
CNS	Central nervous system
CTA	Cancer testis antigen
Cxorf67	Chromosome X open reading frame 67
DMG	Diffuse midline glioma
DSB	Double-strand break
EED	Embryonic ectoderm development
EPN-PFA	Posterior fossa ependymoma subtype A
ESCs	Embryonic stem cells
ESS	Endometrial stroma sarcoma
EZH2	Enhancer of zeste 2 polycomb repressive complex 2 subunit
EZHIP	Enhancer of zeste homologs inhibitory protein
GCs	Germ cells
H3K27M	H3 lysine 27 to methionine
H3K27me3	H3 lysine 27 trimethylation
hNPCs	Human neural progenitor cells
HR	Homologous recombination
IDP	Intrinsically disordered protein
IG	Intronless gene
KLP	K27M-like peptide
MB	Medulloblastoma
MCC	Merkel cell carcinoma
NSCLC	Non-small-cell lung cancer
OS	Osteosarcoma
PALB2	Partner and localizer of BRCA2
PRC2	Polycomb repressive complex 2
PROTACs	Proteolysis targeting chimera
SAM	S-adenosyl methionine
SNVs	Single nucleotide variants
TCA	Tricarboxylic acid
TME	Tumor microenvironment
TULIP	Type B ultra long-range interactions in PFA
