# Supplementary material for: EZHIP in Pediatric Brain Tumors: From Epigenetic Mimicry to Therapeutic Vulnerabilities
Source: Int J Mol Sci. 2026 Jan 18;27(2):963. doi: 10.3390/ijms27020963 (PMC12842176; doi:10.3390/ijms27020963)
Supplement: Supplementary file 1 [file ijms-27-00963-s001.zip › ijms-4043237-supplementary.pdf]

Supplementary Materials

EZHIP in Pediatric Brain Tumors: From Epigenetic Mimicry to Therapeutic Vulnerabilities

Tiziana Servidei [1,*], Serena Gentile [1], Alessandro Sgambato [2,3] and Antonio Ruggiero [1,4,*]

[1] Pediatric Oncology Unit, Fondazione Policlinico Universitario Agostino Gemelli IRCCS, 00168 Rome, Italy; serena.gentile@unicatt.it
[2] Department of Translational Medicine and Surgery, Università Cattolica del Sacro Cuore, 00168 Rome, Italy; alessandro.sgambato@unicatt.it
[3] Multiplex Spatial Imaging Facility, Fondazione Policlinico Universitario Agostino Gemelli IRCCS, 00168 Rome, Italy
[4] Department of Woman and Child Health and Public Health, Università Cattolica del Sacro Cuore, 00168 Rome, Italy
* Correspondence: tiziana.servidei@guest.policlinicogemelli.it (T.S.); antonio.ruggiero@unicatt.it or antonio.ruggiero@policlinicogemelli.it (A.R.); Tel.: +39-06-3015-9967 (T.S.); +39-06-3015-8301 (A.R.)

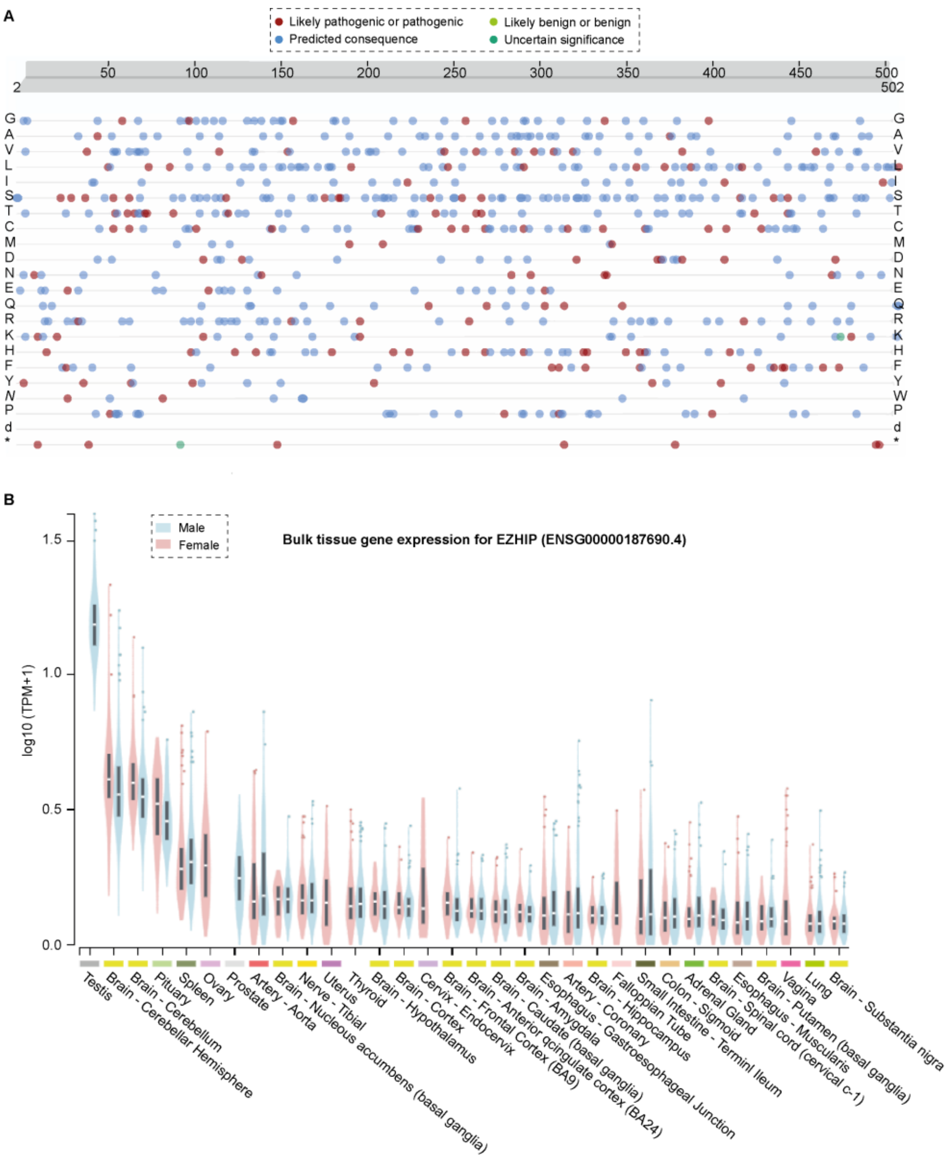

Figure S1. (**A**) Missense single nucleotide variants span throughout the entire EZHIP gene and across a variety of tumors (https://www.uniprot.org/uniprotkb/Q86X51/variant-viewer). (**B**) Bulk tissue gene expression (https://gtexportal.org/home/gene/EZHIP#geneExpression).

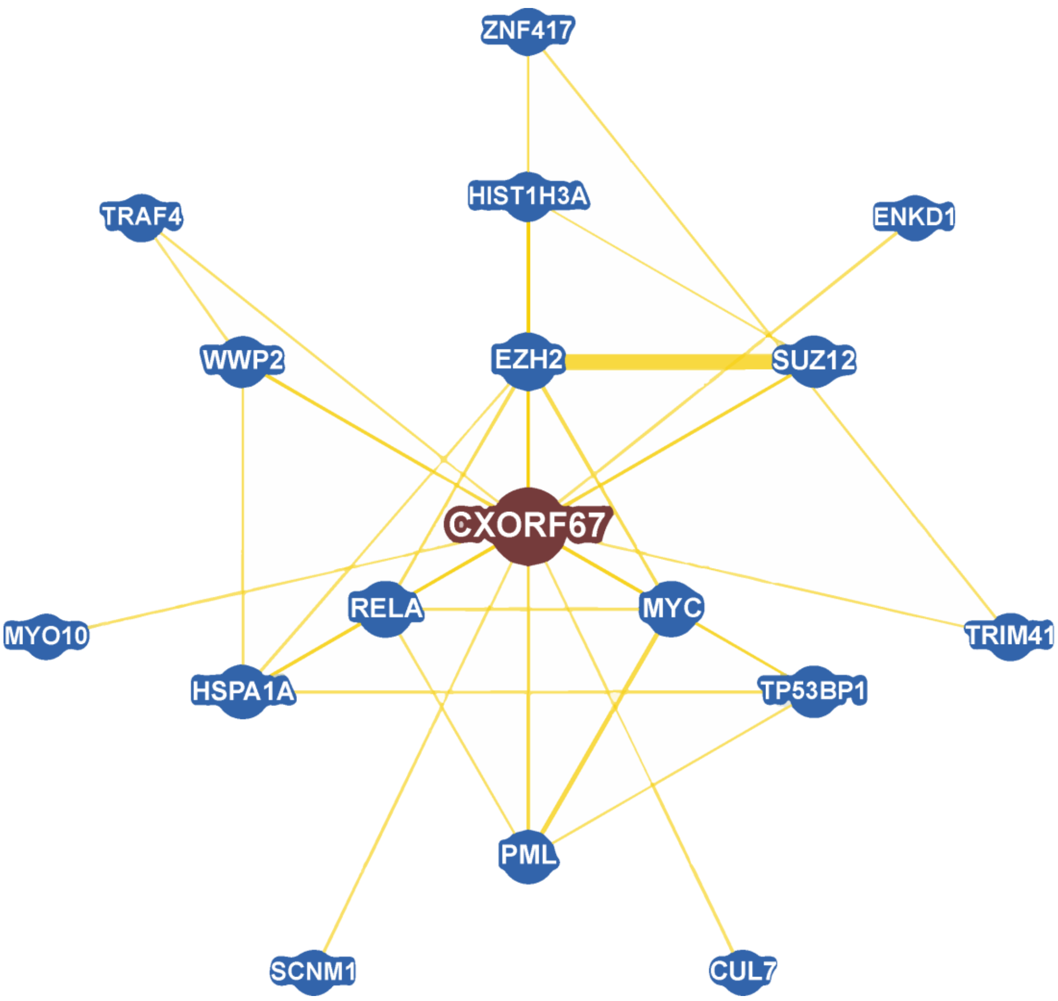

Figure S2. Cxorf67 (EZHIP) interaction network (https://thebiogrid.org/131086/summary/homo-sapiens/cxorf67.html).

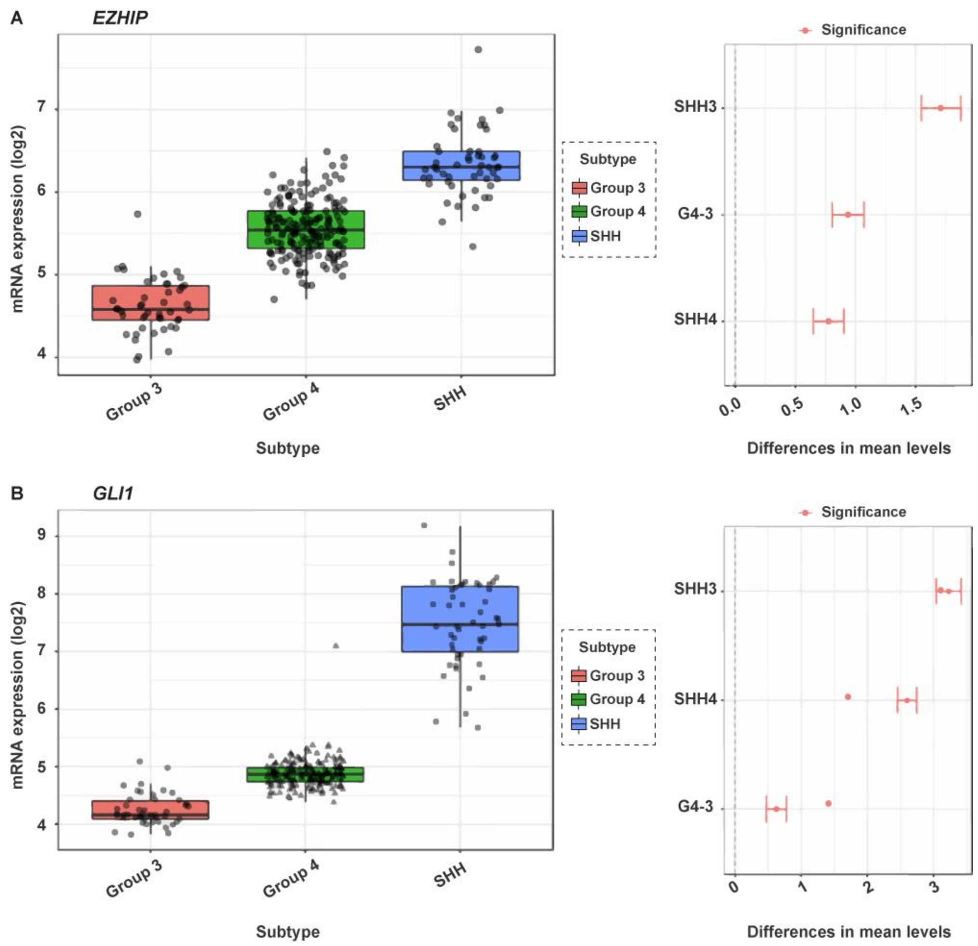

Figure S3. (A, B left) *EZHIP* and *GLI1* mRNA expression across three medulloblastoma subtypes (pediatric cohort, Northcott, 2012). (A, B right) Pairwise comparisons showing differences in mean expression with 95% confidence intervals. (https://gliovis.bioinfo.cnio.es).
